# *Muribaculum intestinale* in brain-gut axis regulation: promises and limitations for therapeutic applications

**DOI:** 10.3389/fmicb.2026.1723051

**Published:** 2026-02-20

**Authors:** Ruijun Wang, Zhanbiao He, Zhiqi Li, Yuanming Pan, Shuang Bai

**Affiliations:** 1 Department of Neurosurgery, Affiliated Hospital of Inner Mongolia Medical University, Hohhot, Inner Mongolia, China; 2 Cancer Research Center, Beijing Chest Hospital, Beijing Tuberculosis and Thoracic Tumor Research Institute, Capital Medical University, Tongzhou, Beijing, China; 3 Department of Dermatology, Affiliated Hospital of Inner Mongolia Medical University, Hohhot, Inner Mongolia, China

**Keywords:** gut microbiota, gut-brain axis, muribaculum intestinale, neuroinflammation, short-chain fatty acids

## Abstract

In recent years, the role of gut microbiota in the regulation of the gut-brain axis has garnered increasing attention, with *Muribaculum intestinale* (*M. intestinale*) emerging as a novel member of the gut microbiota, exhibiting unique biological characteristics and potential therapeutic value. This article systematically reviews the regulatory mechanisms of *M. intestinale* in the gut-brain axis and its associations with various diseases. *M. intestinale* modulates host neurotransmitter synthesis, immune responses, and intestinal barrier function through metabolites such as short-chain fatty acids, succinate, and 3-hydroxybutyrate, thereby influencing the progression of neurodegenerative disorders, psychiatric diseases, and metabolic diseases. Additionally, this article explores the distribution differences of *M. intestinale* in the intestines of mice and humans, as well as its susceptibility to external factors like diet, antibiotics, and exercise. Although current research has unveiled the potential roles of *M. intestinale*, its clinical translation still faces challenges such as technical bottlenecks and individual variability. Future studies should focus on humanized model construction, synthetic biology modifications, and multi-target intervention strategies to achieve precise microbiota-targeted therapies.

## Graphical Abstract

**Figure:**
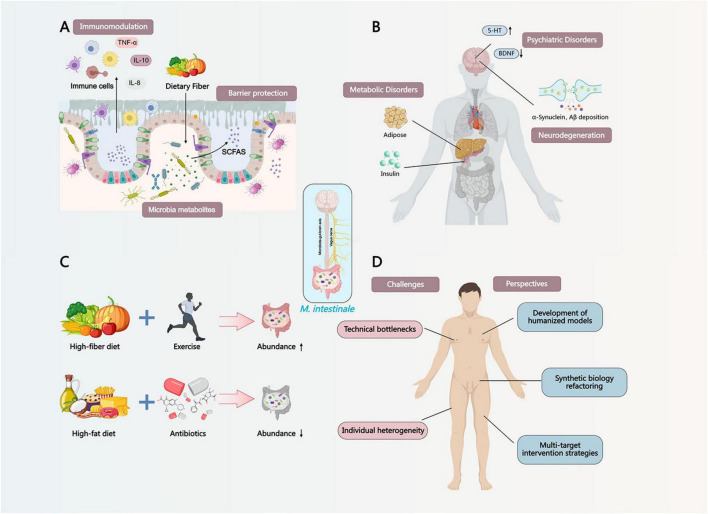

## 1 Introduction

### 1.1 Gut-brain axis and microbial modulation

#### 1.1.1 Multiple signaling pathways including neural, immune, and endocrine

The human body is a complex physiological system where the nervous system, immune system, and endocrine system play crucial roles in maintaining homeostasis and health. The nervous system regulates various physiological activities through rapid electrochemical signaling; the immune system identifies and eliminates foreign invaders to protect the body from diseases; the endocrine system influences functions such as growth, development, metabolism, and reproduction through hormonal regulation.

**FIGURE 1:**
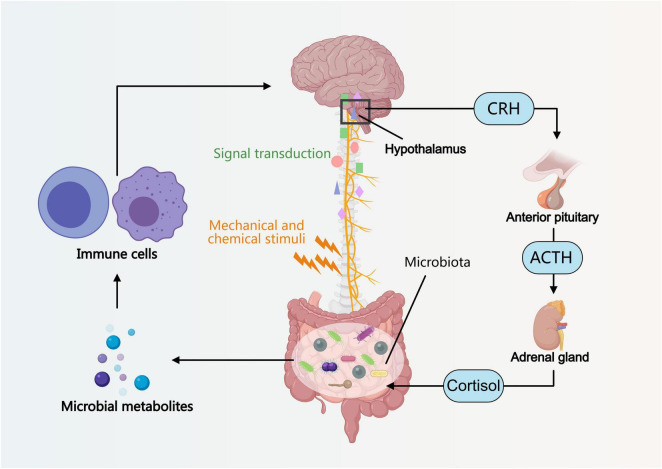
Introduction to the basic concept and bidirectional regulatory mechanisms of the gut-brain axis. Gut microbiota influences brain function through metabolites, thereby affecting the related hormonal regulation of the hypothalamic-pituitary-adrenal axis and the modulation of the vagus nerve.

The gut-brain axis refers to a bidirectional communication system between the brain and the gut, which is not a single pathway but rather a complex interactive network involving multiple signaling pathways such as neural, immune, and endocrine (Figure 1).

In terms of neural pathways, the vagus nerve serves as a crucial bridge connecting the brain and the gut, capable of transmitting information from the gut to the brain while also relaying the brain’s commands to the gut (Sibai et al., 2020; Longo et al., 2023; Lyu et al., 2024; Hwang and Oh, 2025; Wang Y. et al., 2025). Mechanical or chemical stimuli within the gut can influence the activity of gut microbiota via the vagus nerve (Bonaz et al., 2018; Cryan et al., 2019; Han et al., 2022; Siopi et al., 2023; Faraji et al., 2025).

In terms of immune pathways, gut microbiota can regulate the host’s immune response through their metabolites (such as short-chain fatty acids), thereby influencing brain function (Galland, 2014; Peirce and Alviña, 2019; Sibai et al., 2020; Archana Gupta et al., 2024). Short-chain fatty acids can affect the activity of immune cells and modulate the release of inflammatory factors, consequently impacting the function of the nervous system (Wenzel et al., 2020; Shi et al., 2021; Ney et al., 2023). In the endocrine pathway, the hypothalamic-pituitary-adrenal (HPA) axis plays a key role in stress responses (Oyola and Handa, 2017; Juruena et al., 2020; Son et al., 2022; Knezevic et al., 2023; Mbiydzenyuy and Qulu, 2024; Sharan and Vellapandian, 2024). Under stress, the HPA axis is activated, leading to increased hormone secretion, which in turn alters the gut environment and microbial composition (Rosin et al., 2021; Yamaoka et al., 2022; Rusch et al., 2023; Tofani et al., 2025). These changes can also affect the brain’s stress response and emotional state.

Collectively, multiple signaling pathways such as neural, immune, and endocrine systems collectively form the bidirectional regulatory network of the brain-gut axis, through which the brain and gut mutually influence and regulate each other, maintaining the body’s physiological balance (Figure 1).

#### 1.1.2 The role of gut microbiota in the gut-brain axis

The gut microbiota plays a crucial role in the gut-brain axis, influencing the host’s physiological functions through various pathways. Firstly, gut microbes can produce multiple metabolites, such as short-chain fatty acids and neurotransmitters, which can directly or indirectly affect brain function (Sibai et al., 2020; Fried et al., 2021; Panther et al., 2022; Frerichs et al., 2024; Kang et al., 2024; Kim N. Y. et al., 2024). Secondly, gut microbiota plays a vital role in maintaining the integrity of the intestinal barrier. A healthy intestinal barrier prevents harmful substances from entering the bloodstream, thereby reducing potential harm to the brain (Li G. et al., 2022; Ou et al., 2023; Conn et al., 2024; Zhang C. et al., 2025). Additionally, gut microbiota can influence the host’s immune system and neurotransmitter levels, further regulating brain activity (Strandwitz, 2018; Gao et al., 2020; Sibai et al., 2020; Kraimi et al., 2024; Loh et al., 2024; Sathyasaikumar et al., 2024; Patel et al., 2025).

Numerous studies have confirmed that certain specific bacterial strains, such as *Lactobacillus* and *Bifidobacterium*, can improve anxiety, depression, and cognitive function (Barros-Santos et al., 2020; Stenman et al., 2020; Rode et al., 2022; Ruiz-Gonzalez et al., 2025). These probiotics can regulate the balance of gut microbiota and produce beneficial metabolites, thereby enhancing the host’s mood and cognitive function. Although extensive research has focused on common probiotics, many potential functional strains remain underexplored. For instance, *M. intestinale*, as an emerging subject of study, is gradually gaining attention for its mechanisms of action in the gut-brain axis and potential applications (Chen L. et al., 2024; Zhang et al., 2024; Zhang Z. et al., 2025).

### 1.2 *M. intestinale*: taxonomy, ecological niche, and known metabolic functions

#### 1.2.1 Distribution characteristics and colonization status in microbiota

**FIGURE 2:**
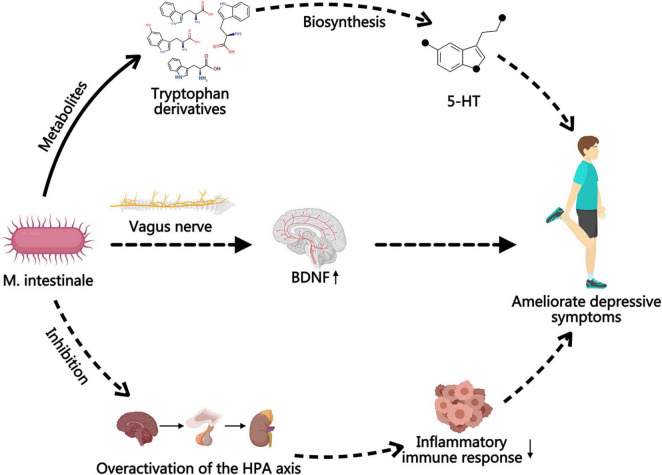
Natural ecological distribution and colonization characteristics of *M. intestinale*. It is one of the priority colonizing strains in mice and may not constitute a core human gut microbiota, but it can maintain long-term colonization at low abundance in individual intestinal microenvironments.

*M. intestinale* belongs to the family *Muribaculaceae* and is a type of obligate anaerobe (Zhu et al., 2024). These bacteria were initially considered part of the S24-7 clade or *Candidatus Homeothermaceae* (Zhu et al., 2024). *M. intestinale* is typically found in relatively high abundance in the intestinal tracts of mice (Dobranowski et al., 2019; Leigh et al., 2020; Maier et al., 2020; McNamara et al., 2021; Zhang et al., 2022; Lee et al., 2023; Chen W. B. et al., 2024; Lin et al., 2024; Shen et al., 2024; Zhang et al., 2024; Zhao et al., 2024; He Y. et al., 2025), yet its detection frequency in human samples is comparatively low (Leigh et al., 2020; Gryaznova et al., 2022). This interspecies distribution disparity may stem from various factors, including dietary habits, genetic backgrounds, and experimental model selection (Leigh et al., 2020; Figure 2).

The colonization and abundance of *M. intestinale* are regulated by external environmental factors, such as antibiotic use and dietary fiber intake (Dobranowski et al., 2019; Dowden et al., 2020; McNamara et al., 2021; Xia et al., 2023; Li J. et al., 2024; Wang Z. et al., 2025). For example, studies have shown that a high-fat diet may reduce the abundance of *M. intestinale* (Li J. et al., 2024), while dietary fiber supplementation may promote its growth (Xia et al., 2023; Wang Z. et al., 2025). Antibiotic use can also lead to gut microbiota dysbiosis, thereby affecting the colonization and abundance of *M. intestinale* (Dobranowski et al., 2019; He Y. et al., 2025; Figure 2).

#### 1.2.2 Relationship with longevity, cognitive improvement, neuroinflammation suppression, and immune regulation

Animal experimental results indicate a positive correlation between the abundance of *M. intestinale* and lifespan extension from the gut of the long-lived rodent *Spalax leucodon.* This may be related to potential mechanisms such as optimizing energy metabolism or reducing oxidative stress by this bacterium (Sibai et al., 2020).

**FIGURE 3:**
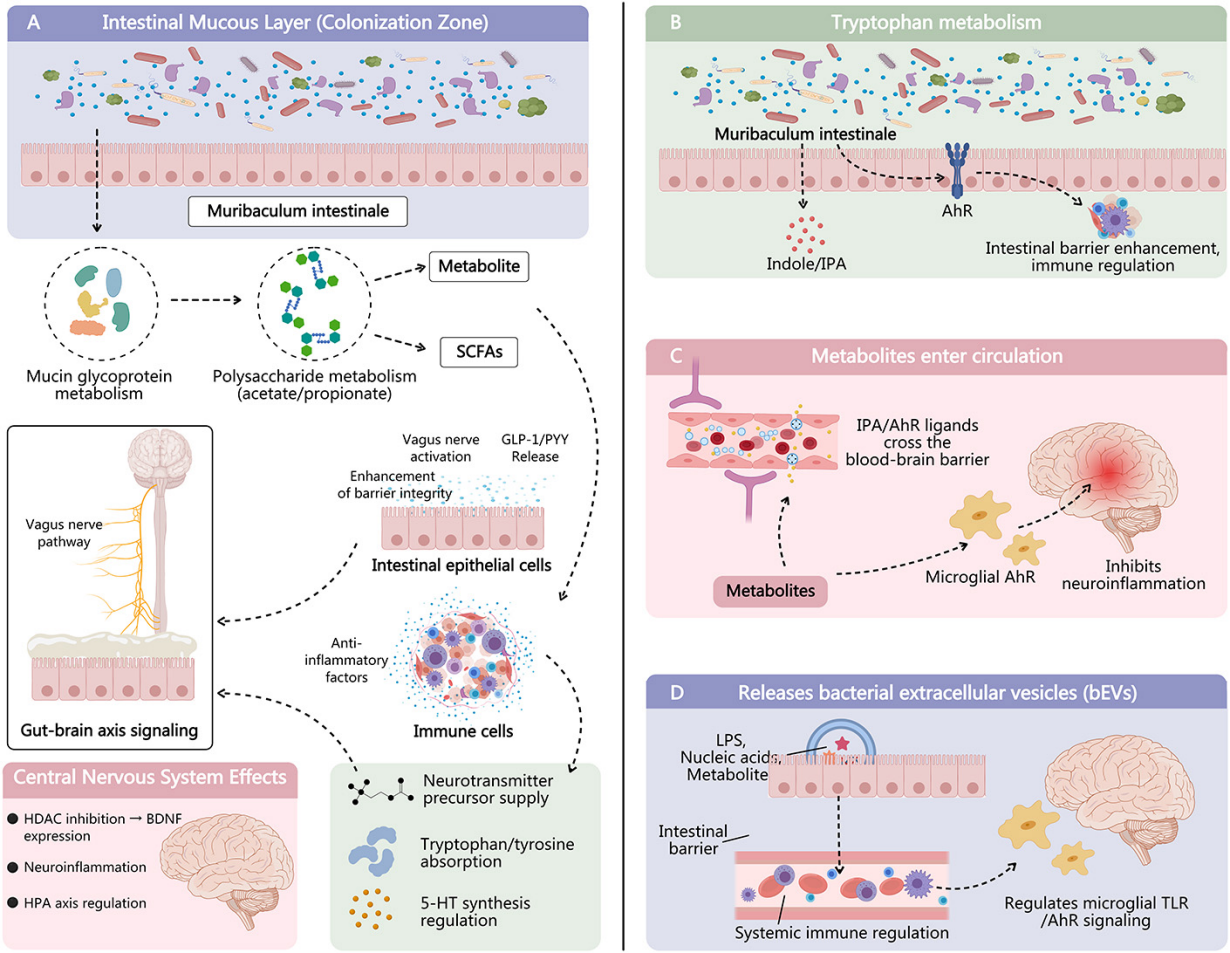
The potential pathways of *M. intestin*ale in gut-brain axis. **(A)** Intestinal mucous layer protection through SCFAs metabolites. **(B)** Tryptophan metabolism, **(C)** Metabolites enter circulation affecting on the brain tissues. **(D)** It possibly releases bacterial extracellular vesicles (bEVs) in brain tissues.

Some research cases indicate that *M. intestinale* can enhance cognitive performance by modulating neurotransmitter synthesis or improving cerebral blood perfusion (Sibai et al., 2020). For example, studies have found that *M. intestinale* can increase levels of brain-derived neurotrophic factor (BDNF) and serotonin (5-HT), thereby improving depressive-like behaviors and cognitive function (Chen L. et al., 2024; Cao et al., 2025; Figure 3).

**FIGURE 4:**
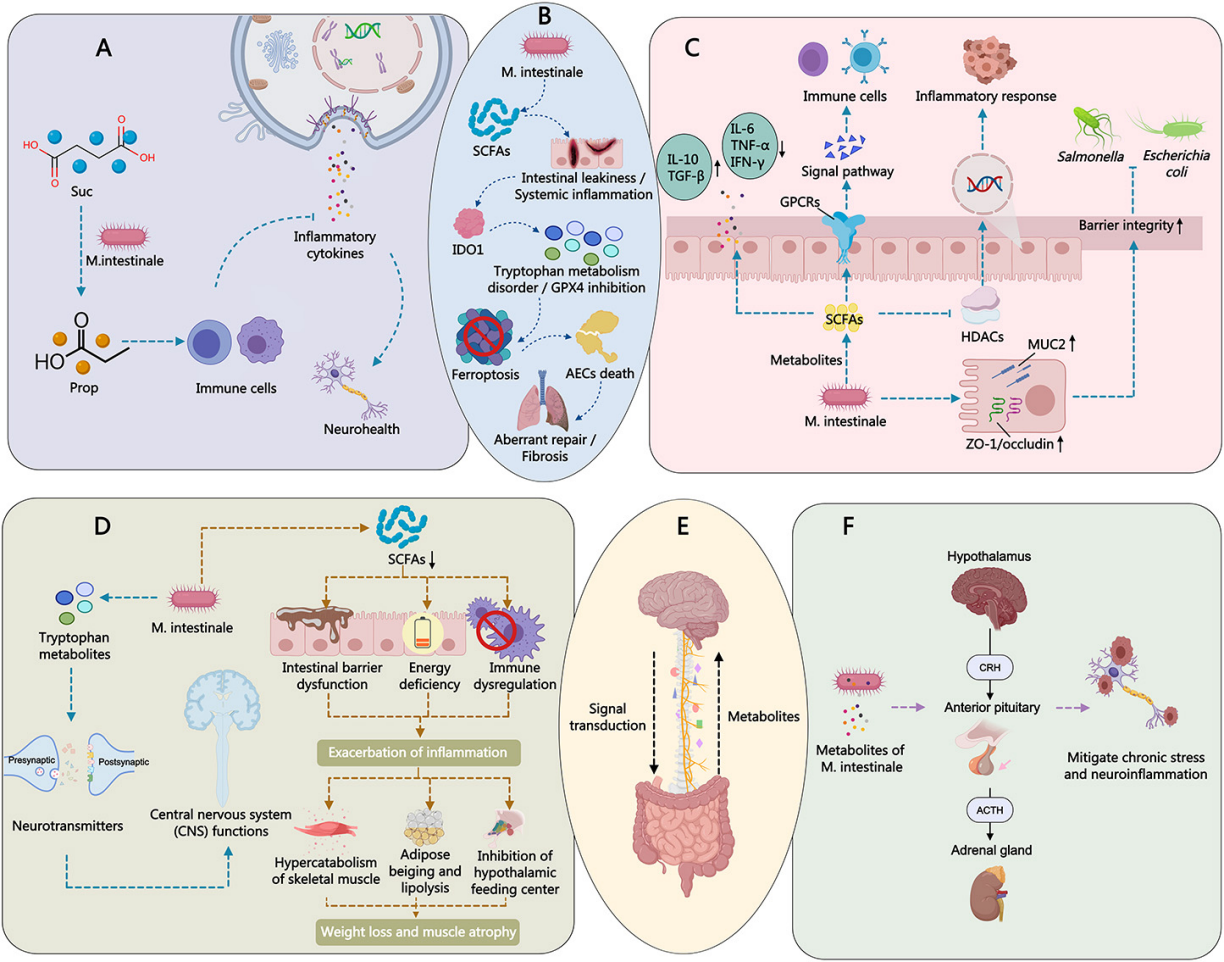
Mechanism of *M. intestinale* improving depressive-like behaviors in depression models by elevating BDNF and 5-HT levels.

The metabolites of *M. intestinale*, such as succinate and short-chain fatty acids, exhibit certain efficacy in inhibiting neuroinflammation (Shen et al., 2024; Cao et al., 2025; Li L. et al., 2025). They can exert anti-inflammatory effects by modulating the expression of pro-inflammatory factors (e.g., IL-6 and TNF-α) and upregulating the expression of anti-inflammatory factors (e.g., IL-10) (Bang et al., 2023; Chang et al., 2026). Additionally, *M. intestinale* can enhance mucosal barrier function or regulate the Th17/Treg cell ratio, thereby exerting a balancing effect on the host immune system (Lee et al., 2023; Hu et al., 2025). For example, studies have found that *M. intestinale* can promote the expression of tight junction proteins and reduce endotoxin (LPS) leakage, thereby resisting systemic inflammation (Zhang et al., 2024; He Y. et al., 2025; Figure 4).

### 1.3 Rationale: knowledge gap on *M. intestinale* in neuroimmune/metabolic pathways

#### 1.3.1 Reveal the molecular mechanism of *M. intestinale* regulating the brain-gut axis

The specific mechanisms of *M. intestinale* in the gut-brain axis are not yet fully understood. This article will provide a comprehensive review focusing on its metabolite production, signaling pathways, and target organ effects to fill this knowledge gap. For example, how does *M. intestinale* influence the synthesis and release of neurotransmitters through metabolic activity? How does it interact with immune cells to regulate inflammatory responses? These are questions worthy of in-depth exploration. Studies by Pérez Escriva et al. have shown that in the gut microbiota of mice, *Bacteroides caecimuris* and *M. intestinale* are the primary suppliers of carbon, with amino acids being the main cross-feeding metabolites (Pérez Escriva et al., 2022). Wang et al. found that *M. intestinale* can convert succinate into propionate, thereby limiting the colonization of *Salmonella typhimurium* (Wang Z. et al., 2025). Chen et al. discovered that 3-hydroxybutyrate derived from *M. intestinale* can alleviate pulmonary fibrosis through IDO1-mediated ferroptosis (Chen et al., 2025; Figure 4B). Bang et al. identified a cardiolipin, MiCL-1, in *M. intestina*le that induces antigen-specific cytokine responses (Bang et al., 2023; Table 1).

**TABLE 1:** Intervention substances and mechanisms of action targeting the potential impacts of *M. intestinale*.

Related additives/interventions	Models	Effects on the gut microbiota	Mechanisms
Hyperforin (Zhang et al., 2022; Zhang Z. et al., 2025)	Anhedonic behaviors in mice	Positively correlated wit*h Akk. muciniphila* and *M. intestinale.*	Hyperforin’s role in modulating gut microbiota metabolism and identifies Carbocysteine as a potential antidepressant.
Flavanones in Citrus unshiu peel (CUP) (Lee et al., 2023)	IBD model with dextran sodium sulfate (DSS) treatment	Increased abundance of *M. intestinale* in the feces of CUP-pretreated group.	CUP improved body weight loss, colon length shortage, and intestinal inflammation than the control mice by decreasing the population of Th17 cells.
Dimethyl itaconate (DI) (He Y. et al., 2025)	*Escherichia coli* (*E. coli*) induced endometritis in mice	FMT from DI-treated group or supplementation of *M. intestinale (DSM 28989*) to recipient mice ameliorated *E. coli*-induced endometritis by promoting the multiplication of *M. intestinale.*	FMT from DI-treated group, or supplementation of *M. intestinale* upregulated the level of guanosine through activating CXCL14 expression in the uterus.
5% *L. paracasei-*fermented and unfermented turmeric (Lin et al., 2024)	High-fat diet (HFD)-induced obese C57BL/6J mice	Enhanced two beneficial bacteria, *Akk. muciniphila and Desulfovibrio*, as well as two SCFA-producing bacteria: *M. intestinale* and *Deltaproteobacteria.*	It suppressed weight gain and liver and visceral adipose tissue weight and reduced plasma metabolic parameters by downregulating the expression of adipogenesis, lipogenesis, and inflammatory-related protein, but upregulated liver β-oxidation protein SIRT 1, PPARα, and PGC-1α in perigonadal adipose tissue.
Gingerols-enriched ginger (GEG) (Shen et al., 2024)	Diabetic neuropathic pain (DNP) rats	GEG increased the abundance of *Acinetobacter, Azospirillum, Colidextribacter, and Fournierella*, but decreased abundance of *M. intestinale* in cecal feces of rats.	GEG decreased pain, anxio-depression, and improved the composition of gut microbiomes and mitochondrial function.
Selenium (Li J. et al., 2024)	Mice with breast cancer under a high-fat diet	An increased abundance of *Proteobacteria, Actinobacteria*, and *Verrucomicrobia;* Species such as *Helicobacter ganmani, Helicobacter japonicus*, and *Akk. muciniphila*, while phyla, such as *Bacteroidetes, Firmicutes, Deferribacteres*, and *Spirochaetes*, including decreased species, such as *Prevotella* sp. *MGM2, M. intestinale, Lactobacillus murinus*, and *Prevotella* sp. *MGM1.*	Alters the homeostasis of gut microbiota in mice with breast cancer on a high-fat diet by interfering with gut microbiota homeostasis, leading to altered synthesis of tumor-associated proteins and fatty acids and inducing tumor cell apoptosis and pyroptosis.
Arbutin (AR), a natural glycoside compound exerting neuroprotective properties (Cao et al., 2025)	Chronic unpredictable mild stress (CUMS) mice	AR alleviated the depressive-like behaviors, inflammatory reaction, oxidative stress, restored neurotrophic factors and gut tight junction proteins	TLR4/NF-κB/IRAK1 signaling was involved in these alterations. AR enriched *muribaculaceae* and tryptophan metabolism. *M. intestinale*. AR ameliorated depressive-like behaviors, inflammation and 5-HT content, Tryptophan Hydroxylase 1 (TPH1) and Indoleamine 2,3-Dioxygenase 1 (IDO1) expressions, tryptophan metabolism and kynurenine route. The molecular docking and molecular dynamic suggested that AR might bind to TPH1 and IDO1.
Sargassum fusiforme-derived exosome-like nanoparticles (SELNs) (Chang et al., 2026)	Citrobacter rodentium-induced colitis	SELNs exhibited prolonged gut retention and colon-targeting efficacy and alleviated colitis symptoms, including body weight loss, colonic shortening, and histological damage	SELNs promoted tight junction and MUC2 protein expression and suppressed the TLR4/MyD88/NF-κB cascade, resulting in downregulated pro-inflammatory cytokines (TNF-α, IL-6, IL-1β) and upregulated anti-inflammatory cytokines (IL-10, IL-22), coupled with iNOS abrogation. Furthermore, SELNs modulated the gut microbiota composition, especially increasing the abundance of *M. intestinale*, and promoted the production of SCFAs.
Heterophyllin B (Chen et al., 2025)	Mice with bleomycin (BLM)-induced pulmonary fibrosis (PF)	The antifibrotic efficacy of HB was contingent on the enrichment of *M. intestinale* and its metabolite, 3-hydroxybutyrate	HB alleviates PF by eliminating intestinal microecology and suppressing IDO1-mediated ferroptosis
Ginsenoside Rd (G-Rd) (Liu et al., 2025)	Metabolism-associated fatty liver disease (MAFLD) model	G-Rd significantly reduced liver injury and steatosis in MAFLD mice and downregulated the elevated abundance of *Firmicutes* and the *Firmicutes/Bacteroidetes* ratio. It also significantly reduced the abundances of *Faecalibaculum rodentium* while increasing *M. intestinale*, with its functional role being relevant to lipid metabolism regulation.	G-Rd ameliorated mitochondrial damage and inhibited the ferroptosis pathway in the liver mediated by Nrf2 signaling
Ginseng Polysaccharides (GPs) (He Y. et al., 2025)	*Aspergillus sydowii*-driven Lung Adenocarcinoma model	GPs significantly inhibited *A. sydowii*-induced tumor growth in both models by enriching *Lactobacillus/M. intestinale* and suppressing pro-inflammatory *Alistipes*	GPs exert antitumor effects against A. sydowii-induced LUAD by modulating gut microbiota and bile acid metabolism
Cranberry polyphenols (CP), rich in flavonoids, and agavins (AG), a highly branched agave-derived neo-fructans (Medina-Larqué et al., 2022)	C57BL6 male mice fed an obesogenic high-fat and high-sucrose (HFHS) diet	AG, either alone or combined with CP (CP + AG), stimulated the glycan-degrading bacteria *M. intestinale*, *Faecalibaculum rodentium*, *Bacteroides uniformis*, and *Bacteroides acidifaciens*.	CP + AG-supplemented HFHS-fed mice had significantly lower levels of plasma LBP than HFHS-fed controls, increasing TLR-2 expression, while decreasing the expression of ILβ1 in obese mice.
Chaigui granules (CGG), a novel traditional Chinese medicine formulation for depression (Wu et al., 2026)	Murine FMT-induced depression models	CGG inhibited *Bacteroides caccae*, *Clostridium cocleatum*, and *M. intestinale*, while promoting *Akk. muciniphila* and *Mucispirillum schaedleri*.	Chaigui granules alleviate depressive-like behavior in murine FMT-induced depressed mice by modulating Aminoacyl-tRNA biosynthesis and Lysine degradation.
Total flavonoids of Chrysanthemum indicum L (TFC) (Yang et al., 2024)	Acute pancreatitis models (AP)	Increased *Bacteroides sartorial, Lactobacillus reuteri, M. intestinale*, and *Parabacteroides merdae* by AP, and decrease of *Helicobacter rodentium, Pasteurellaceae bacterium*, Streptococcus hyointestinalis by AP were both reversed by TFC treatment.	TFC can effectively suppress AP progression and AP induced colonic barrier dysfunction by mitigating elevated serum amylase, MPO levels.
Combination of antibiotics and the AAD diet (Quinn et al., 2020)	*H. pylori*-infected male INS-GAS mice	Reduction of menA from *Akk. muciniphila*, *Bacteroides uniformis*, and *M. intestinale* were confirmed in antibiotic-treated mice.	Antibiotic therapy decreased gastric pathology, but dietary folate had no effect. However, the combination of antibiotics and the AAD diet induced anemia, gastric hemorrhage, and mortality, leading to Vitamin K deficiency.

#### 1.3.2 Exploring its application prospects in neurodegenerative, psychiatric, and metabolic diseases

Based on the aforementioned content, it is predicted that *M. intestinale* may play a significant role in future precision intervention strategies for diseases such as Alzheimer’s disease, depression, and obesity. For instance, could cognitive function in Alzheimer’s patients be improved by modulating the gut microbiota to increase the abundance of *M. intestinal*e? Could the metabolites of *M. intestinale* serve as novel antidepressant drugs? These are directions worthy of further research. Studies by Zhang et al. showed that hypericin-induced gut microbiota metabolite kaposi cysteine protects mice from depression-like behaviors by regulating the colonic mucus barrier, with kaposi cysteine being associated with beneficial bacteria *Akkermansia muciniphila* and *M. intestinale* (Zhang Z. et al., 2025). Chen et al. found that SFN and its derivatives can modulate the composition of gut bacteria, including *Firmicutes*, *Actinobacteria*, *Parabasalia*, and *Tenericutes*, as well as *Bacteroidales* bacteria, *Lachnospiraceae bacteria A4*, *Muribaculum intestinale*, *Muribaculaceae bacteria*, and *Prevotella* sp. *MGM1*, potentially altering their functions related to anti-inflammatory effects (Chen L. et al., 2024). Lin et al. demonstrated that fermented turmeric can regulate gut microbiota composition, particularly two beneficial bacteria, *Akkermansia muciniphila* and *Desulfovibrio*, as well as two short-chain fatty acid-producing bacteria: *M. intestinale* and *Deltaproteobacteria* (Lin et al., 2024).

### 1.4 Review objectives and PECO statement

Based on the statement of PECO listed as follows: (1) Population: Experimental animal models (mice/rats); (2) Exposure: Administration of M. intestinale (live, inactivated, or its derivatives); (3) Comparator: Control groups without M. intestinale intervention; (4) Outcomes: Behavioral, neuroinflammatory, metabolic, or microbiota composition changes. This paper aims to integrate the latest research findings to provide a reference framework for understanding the biological functions of *M. intestinale* and its regulatory role in the gut-brain axis, while laying a theoretical foundation for the development of novel microecological therapies.

## 2 Materials and methods

### 2.1 Literature search strategy

A systematic MEDLINE search for all publications referring to *M. intestinale* was selected using PubMed by English language filter. We included all eligible included articles and reviews up to 1st January 2015. For the search, the relative Medical Subject Heading (MeSH) terms were used. All data were collected, screened and referenced in accordance with the PRISMA guidelines.

### 2.2 Inclusion and exclusion criteria

All papers were reviewed for inclusion by two authors. When there was uncertainty or conflict in decisions, it was discussed to reach a consensus. Articles eligible to be included in this review were required to meet the following criteria: *Inclusion*: (1) *In vivo* animal studies (any species/strain/age); (2) Interventions involving M. intestinale (mono-colonization, probiotic, postbiotic); (3) Measurable outcomes related to GBA (e.g., behavioral tests, cytokine levels, gut permeability, microbiome shifts); (4) Published in peer-reviewed journals, no language or date restrictions. *Exclusion*: *In vitro* or *ex vivo* studies alone; Studies without a control group; Reviews, conference abstracts without full data; Studies focusing solely on gastrointestinal outcomes without neural/behavioral measures.

### 2.3 Information sources and search strategy

(1)    Databases: PubMed, Web of Science, Embase, Scopus.
(2)    Gray literature: OpenGrey, preprint servers (bioRxiv, medRxiv).
(3)    Search terms: (“*Muribaculum intestinale*” OR “*Muribaculum”*) AND (“gut-brain axis” OR neuro* OR behav* OR cognit* OR anxiet* OR depress* OR “short-chain fatty acids” OR microglia OR “blood-brain barrier”).
(4)    Timeframe: Inception to (2015.01-2026.02).

### 2.4 Study selection process

(1)    Two independent reviewers (screening by title/abstract → full-text).
(2)    Discrepancies resolved by a third reviewer.
(3)    PRISMA flow diagram detailing identification, screening, eligibility, inclusion.

### 2.5 Data extraction

Extracted items:

1. Study characteristics (author, year, country, funding)
2. Animal model details (species, strain, age, sex, housing)
3. Intervention (strain source, dosage, duration, administration route)
4. Outcomes (primary/secondary endpoints, measurement methods, timepoints)
5. Key findings (statistical results, direction of effect)

### 2.6 Assessment of risk of bias

The methodological quality of included animal studies was evaluated using SYRCLE’s Risk of Bias tool, which covers ten domains of internal validity. Each domain was judged as “Low,” “High,” or “Unclear” risk of bias. Assessments were performed independently by two reviewers, with disagreements resolved through discussion or consultation with a third reviewer.

### 2.7 Ethical considerations

This systematic review did not involve direct animal experimentation. However, we extracted and reported whether included studies provided statements regarding ethical approval from institutional animal care and use committees (IACUC) and adherence to the ARRIVE 2.0 guidelines. The review process itself followed ethical scholarly practices, ensuring transparent reporting and unbiased synthesis.

## 3 Results

### 3.1 Taxonomic status and morphological characteristics

#### 3.1.1 Phylogenetic placement

*M. intestinale* belongs to the family *Muribaculaceae* (formerly known as the *S24-7* group or *Candidatus Homeothermaceae*) within the phylum *Bacteroidetes*. Members of this family primarily colonize the cecum and colon of rodents and are one of the dominant bacterial strains commonly found in the mouse gut microbiota (Zhu et al., 2024). Phylogenetic analysis reveals that although *M. intestinale* shares the same phylum *Bacteroidetes* with typical gut genera such as *Prevotella* and *Bacteroides*, it forms an independent branch. Its 16S rRNA gene sequence typically exhibits less than 95% similarity to other genera within the same family, reflecting its evolutionary uniqueness (Zhu et al., 2024). Strains of the *Muribaculaceae* family generally possess extensive polysaccharide utilization capabilities, encoding a large number of carbohydrate-active enzymes (CAZymes) in their genomes, particularly glycoside hydrolase gene clusters (e.g., SusC/D homologs) associated with plant polysaccharide and host mucin degradation (Pérez Escriva et al., 2022). These genomic features endow *M. intestinale* with ecological adaptability in response to fluctuations in complex carbon source availability, enabling rapid proliferation and sustained colonization in high-fiber environments or mucus-rich niches.

#### 3.1.2 Morphology and basic characteristics such as anaerobic, gram-negative

In terms of morphology, *M. intestinale* appears as a Gram-negative straight rod, typically measuring approximately 0.5–1.0 μm in diameter and 2–3 μm in length, without spore formation, and no flagella or capsule structures have been observed (Hwang and Oh, 2025). As a strict anaerobe, *M. intestinale* is highly sensitive to oxygen and easily loses viability in normoxic environments, thus requiring isolation and cultivation under conditions with oxygen levels below 0.1%, typically using anaerobic jars or atmosphere-controlled systems with complex media containing intestinal secretions or reducing substrates. Its Gram-negative cell wall is rich in LPS, where the lipid A structure and polysaccharide chain length play a dual regulatory role in stimulating host immune responses and maintaining intestinal barrier stability: on one hand, it can trigger low-level homeostatic immune activation via the TLR4 pathway, while on the other, it may induce inflammatory responses during microbial dysbiosis.

### 3.2 Ecological distribution and colonization characteristics

#### 3.2.1 Abundance and presence in the intestinal tracts of mice and humans

In commonly used laboratory mouse strains such as C57BL/6J, *M. intestinale* is often one of the preferentially colonizing strains in the cecum and colon. In the Oligo-Mouse Microbiota (OMM) model, it is listed alongside *Bacteroides caecimuris* as a primary carbon source provider, reflecting its important role in the murine gut niche (Pérez Escriva et al., 2022). Traditional metagenomic sequencing reveals that in wild-type and knockout (e.g., AC5KO) mice, the abundance of *M. intestinale* can reach 5–15% of the total gut microbiota, demonstrating localized colonization advantages, particularly enriched in cecal contents (Pérez Escriva et al., 2022). In contrast, the detection rate and abundance of *M. intestinale* in the human gut are generally significantly lower, with relative abundances often below 0.1% in most metagenomic studies. Only in specific populations or under special dietary interventions (e.g., Mediterranean diet, plant-based high-fiber intake) are minor increases observed, suggesting it may not constitute a core human gut microbiota but can persist at low abundance in individual gut microenvironments (Gryaznova et al., 2022; Figure 2).

#### 3.2.2 Regulation of colonization and abundance by external factors

Dietary structure is one of the key factors influencing the abundance of *M. intestinale*. Multiple studies indicate that high-fiber or polysaccharide-rich diets significantly promote its growth: for instance, Japanese traditional koji amazake (a fermented rice drink prepared via disaccharide conversion) markedly increases the relative abundance of *M. intestinale* in mouse cecum, while control groups maintain lower levels (Murakami et al., 2024). Similarly, cranberry polyphenol combined with agave oligosaccharide supplementation (CP + AG) selectively stimulates the expansion of mucin-degrading bacteria represented by *M. intestinale* under high-fat, high-sugar conditions, accompanied by elevated butyrate levels and improved gut barrier function (Medina-Larqué et al., 2022; Table 1). Conversely, high-fat or low-fiber Western diets persistently suppress *M. intestinale* abundance-even a single juvenile Western diet intervention can sustain low abundance into adulthood in mice, suggesting early dietary patterns exert a “programing” effect on its long-term colonization (McNamara et al., 2021; Table 2).

**TABLE 2:** Observation of different models affecting the abundance of *M. intestinale*.

Models	Variations of gut microbiota
Blind mole-rat (*Spalax leucodon*) with longevity (Sibai et al., 2020)	The longevity-linked *Muribaculaceae* bacterial family was detected obviously.
Sleep deprivation (SD)-induced anxiety-like behaviors in rats (Zhang et al., 2024)	After SD, the richness of *Akk. muciniphila*, *M. intestinale* and *Bacteroides caecimuris* was decreased.
SHIP^–/–^ mice after the onset of overt IBD (Dobranowski et al., 2019)	Reduction in the *Bacteroidales taxa*, *M. intestinale* and the expansion in *Lactobacillus.*
Juvenile Western diet in early-life model for 8 weeks (McNamara et al., 2021)	Bacterial richness, diversity and the abundance of *M. intestinale* were reduced.
*Lacticaseibacillus rhamnosus Probio-M9* (Zhao et al., 2024)	A protective effect in a colitis-associated cancer (CAC) model by regulating key bacteria *(*including *Lactobacillus murinus, Muribaculaceae bacterium DSM 103720, M. intestinale* and *Lachnospiraceae bacterium A4.*
Cachectic mice (Li L. et al., 2025)	A significant alteration in gut microbiota composition, particularly a reduction in *M. intestinale (82.0%).* Direct supplementation with *M. intestinale* increased its abundance and butyrate level reduced muscle wasting in cachexia. Positive correlation between *M. intestinale* and butyrate level.
Double saccharification of Koji amazake, made from rice and rice koji (Murakami et al., 2024)	Health benefits with the enrichment of *Anaerotignum lactatifermentans*, *M. intestinale*, and *Parabacteroides merdae.*
*C. difficile* in an antibiotic-treated mouse model (Foley et al., 2025)	Using *M. intestinale* restricts *C. difficile*’s growth, playing a role in establishing colonization resistance.
Ampicillin in maternal intrapartum antibiotic prophylaxis or postpartum maternal antibiotic usage (Zuffa et al., 2025)	Depletion of beneficial bacterial species belonging to the *Muribaculaceae* family, including *M. intestinale* and *Duncaniella dubosii*, and led to cohort-dependent enrichments of *Enterococcu* and *Prevotella* species.
Avian coccidiosis induced by *E. maxima* (Xue et al., 2025)	Increased the abundance of bacterial species, including *Clostridioides difficile*, *Faecalibacterium prausnitzii*, *Mediterraneibacter torques*, *M. intestinale*, *Mediterraneibacter massiliensis*, *Phascolarctobacterium faecium*, and *Phocaeicola plebeius.*

The interaction between exercise and host genotype also influences the selective colonization of *M. intestinale*. Analysis of gut microbiota in AC5KO mice under both exercise and non-exercise conditions revealed species-level selective distribution of *M. intestinale* alongside *Parasutterella excrementihominis*, with its abundance upregulated during exercise, suggesting that physical activity may promote colonization by altering gut transit time, mucus secretion, or immune homeostasis (Dowden et al., 2020). Additionally, chronic stress and sleep deprivation experiments demonstrated that sustained stress reduces the relative abundance of *M. intestinale*, accompanied by elevated serum LPS levels and anxiety-like behaviors, implying its protective role in the stress-gut-brain axis (Zhang et al., 2024; Table 2).

The application of antibiotics has a significant impact on *M. intestinale*, which is highly sensitive to various commonly used antibiotics (such as metronidazole, vancomycin, etc.), almost completely disappearing upon use. Full recovery requires fecal microbiota transplantation or several weeks, and in germ-free mice, it is difficult to recolonize without microbiota reconstruction (He Y. et al., 2025).

### 3.3 Metabolic function and microbial interactions

#### 3.3.1 Metabolite productions

*M. intestinale* acts as an “intermediate supplier” in the carbon metabolic network, with its fermentation products primarily consisting of dicarboxylic acids (such as succinate) and short-chain fatty acids (SCFAs). In *in vitro* co-culture experiments with OMM-synthesizing bacterial consortia, *M. intestinale* produces high levels of succinate, becoming the main carbon source exporter in the co-culture system. Succinate can be further converted into butyrate by other bacteria, such as members of the *Clostridia* phylum, promoting anaerobic respiration and intestinal barrier repair (Pérez Escriva et al., 2022). Further studies focus on its unique ability to specifically convert succinate into propionate: research on a murine fiber-deficient model and *Salmonella enterica* infection demonstrated that oral administration of *M. intestinale* effectively reduces free succinate levels in the intestinal lumen while converting it into propionate. Propionate enhances colonization resistance and suppresses pathogen proliferation through GPR43/GPR41 receptor-mediated anti-inflammatory signaling pathways (Wang Z. et al., 2025; Figure 3A).

Recent studies have also found that in a pulmonary fibrosis model intervened by the natural cyclic peptide Heterophyllin B, *M. intestinale* was significantly enriched, and its secreted 3-hydroxybutyrate (3-HB) plays an important role in regulating IDO1-mediated ferroptosis, helping to alleviate pulmonary fibrosis symptoms, suggesting that *M. intestinale* has the potential to produce small-molecule neuroprotective or anti-fibrotic metabolites under the induction of specific compounds (Chen et al., 2025; Figure 4B).

#### 3.3.2 Potential metabolic pathways

*M. intestinale* has drawn attention due to its enrichment in the intestines of healthy populations and its association with anti-inflammatory effects and butyrate production. However, most studies remain at the level of descriptive correlations (metagenomic association analysis, metabolomics detection) and preliminary functional validations (Tables 1, 2). Research on the molecular mechanisms governing its synthetic metabolic pathways, such as transcriptional regulation, enzyme activity modulation, and environmental signal sensing, is virtually absent.

Based on some promising linkages of *M. intestinale* in gut-brain axis, we have listed the potential roles of *M. intestinale* in tryptophan metabolism, microglial regulation, and bacterial extracellular vesicles by driving the secretion of SCFAs and neurotransmitter-like molecules (Figure 3).

##### 3.3.2.1 Tryptophan metabolism

Tryptophan degradation pathway:*M. intestinale* breaks down dietary tryptophan into indole and its derivatives (such as indole-3-propionic acid, IPA) via tryptophanase or related enzymes encoded by its genome (Zhu et al., 2024). Indole compounds can act as ligands for the aryl hydrocarbon receptor (AhR), activating the AhR signaling pathway, regulating intestinal barrier function, immune balance, and neuroinflammation (Li et al., 2021; Wang et al., 2023; Kim H. et al., 2024; Li Q. et al., 2025; Wang X. et al., 2025; Figure 3B).

Immune and neural regulation: Metabolites such as IPA possess antioxidant and anti-inflammatory properties, can cross the blood-brain barrier (BBB), inhibit excessive activation of microglia, and reduce neuroinflammation (Si et al., 2026; Figure 3C). AhR activation can also promote intestinal epithelial cells to secrete IL-22, enhance barrier integrity, and reduce systemic inflammation (Yang et al., 2020; Li Y. Y. et al., 2022; Meynier et al., 2022; Kang et al., 2025).

##### 3.3.2.2 Microbial regulation of microglia

Indirect pathway (through metabolites): Tryptophan metabolites (e.g., IPA) produced by *Muribaculum* enter the central nervous system (CNS) via the bloodstream, directly acting on AhR receptors on microglia, regulating their phenotype (transforming from pro-inflammatory M1 to anti-inflammatory M2). Other SCFAs may indirectly affect microglial functions by inhibiting histone deacetylases (HDAC) or activating G protein-coupled receptors (GPCRs) (Figure 3A; Hosseinkhani et al., 2021; Saadh et al., 2025).

Immune-brain axis signaling: Gut microbiota metabolites indirectly inhibit microglial inflammatory responses by regulating peripheral immune cells (such as Treg cells) to release anti-inflammatory factors like IL-10 and TGF-β (Jiang et al., 2020; Guan et al., 2025; Yong et al., 2025; Allani et al., 2026).

##### 3.3.2.3 The role of bacterial extracellular vesicles

Vesicle contents: bEVs released possibly by *probiotics* carry LPS, peptidoglycans, DNA, RNA, and enzymes, potentially entering the circulation through the intestinal barrier. AhR ligands (e.g., indole) in bEVs can directly activate the AhR pathway in distant cells (including microglia) (Li J. et al., 2024; Figure 3D).

Immune modulation: bEVs can be taken up by intestinal immune cells or cells of distant organs, modulating TLR/NF-κB and other signaling pathways, affecting systemic immune status. In the nervous system, bEVs may activate microglia through TLR4, but their effects depend on the contents (pro-inflammatory or anti-inflammatory) (Ha et al., 2023; Ma et al., 2024).

#### 3.3.2 Synergistic effects with other probiotics and immunomodulatory effects

*M. intestinale* can synergize with various probiotics to form complementary networks of mucus layer degradation or polysaccharide metabolism, further influencing host immunity. In stress-induced mouse models treated with the antidepressant active ingredient hyperforin, both *M. intestinale* and the Gram-negative mucin-producing bacterium *A. muciniphila* were enriched. They collaboratively enhanced intestinal mucus barrier integrity and reduced systemic inflammatory factor TNF-α levels, thereby improving depressive-like and anhedonic behaviors (Zhang et al., 2022). Similarly, in obese mouse models with combined CP and AG administration, the synergistic proliferation of *M. intestinale* and *A. muciniphila* was closely associated with the activation of intestinal Toll-like receptor 2 (TLR2) signaling, promoting anti-inflammatory IL-10 secretion and regulatory T cell expansion, thereby suppressing obesity-related low-grade chronic inflammation (Medina-Larqué et al., 2022). In a colon-associated tumor mouse model, *Lactobacillus Probio-M9* intervention not only repaired Azoxymethane/DSS-induced microbiota disruption but also selectively restored key strains such as *M. intestinale* and *Lactobacillus murinus*, accompanied by the recovery of tight junction protein expression and improvement of the inflammatory microenvironment, indicating that *M. intestinale* also has potential probiotic effects in anti-tumor inflammatory microenvironments (Chen W. B. et al., 2024).

Additionally, the specific cardiolipin (MiCL-1) produced by *M. intestinale* can activate host dendritic cells and T cells through the TLR2/TLR1 complex, inducing the secretion of pro-inflammatory cytokines such as TNF-α, IL-6, and IL-23, thereby providing signaling support for antigen-specific cellular immune responses, suggesting its dual role in trained immunity and microbiota symbiosis homeostasis (Bang et al., 2023). Using techniques like co-culture and isotope tracing, future studies could further elucidate the cross-feeding network of carbon and nitrogen nutrients between *M. intestinale* and other probiotics, as well as their molecular mechanisms in host immune tolerance and defense.

### 3.4 The regulatory mechanism of *M. intestinale* in the gut-brain axis

#### 3.4.1 Metabolic pathways and neural regulatory mechanisms

##### 3.4.1.1 Conversion of succinate to propionate and its anti-inflammatory effects

In the gut microbiota, *M. intestinale* plays a unique metabolic role, and its metabolites may have significant effects on the gut-brain axis. *M. intestinale* can convert succinate into propionate, a process that may involve a series of complex biochemical reactions and key enzymes. As a short-chain fatty acid (SCFA), propionate can exert anti-inflammatory effects through multiple mechanisms (Figure 4A).

Research indicates that propionic acid can inhibit the release of inflammatory factors, thereby reducing inflammatory responses. The specific anti-inflammatory mechanisms include modulating immune cell functions, such as influencing the activity of macrophages and T cells. Through these mechanisms, propionic acid can alleviate inflammation in the gut and brain, thereby improving neurological health. *M. intestinale* plays a significant role in mitigating gut-brain inflammation by converting succinate to propionate (Pérez Escriva et al., 2022; Wang Z. et al., 2025; Figure 4A). For example, *M. intestinale* can limit the colonization of *Salmonella Typhimurium* by converting succinate to propionate. When supplementing with propionate, rather than succinate, which enhances resistance to *Salmonella Typhimurium* colonization in mice fed a fiber-free diet (Wang Z. et al., 2025).

##### 3.4.1.2 Tryptophan metabolism and the regulation of neurotransmitters

*M. intestinale* is involved in tryptophan metabolism, affecting the synthesis and regulation of neurotransmitters, with 5-HT being one of the important neurotransmitters. Tryptophan metabolism primarily proceeds through two pathways: the indole compound pathway and the kynurenine pathway.

Indole compounds are metabolites of tryptophan metabolism and can influence the gut-brain axis by modulating intestinal immunity and neural function. The metabolic products generated by the kynurenine pathway, such as kynurenine and quinolinic acid, have significant effects on the neurotransmitter system. These metabolites can cross the blood-brain barrier and directly or indirectly regulate the function of the central nervous system.

In mouse models, studies have shown that *M. intestinale* can influence behavioral manifestations such as anxiety- and depression-like behaviors by modulating the levels of tryptophan metabolites. These metabolites indirectly regulate the function of the central nervous system by affecting neurotransmitter systems, thereby impacting mood and cognitive functions (Zhang et al., 2024; Zhang Z. et al., 2025). Zhang et al. found that under a high-fat diet, the abundance of *M. intestinale* was associated with anxiety-like behaviors, suggesting its potential role in affecting brain function through the modulation of gut metabolites (Zhang Z. et al., 2025).

### 3.5 Immune regulation and signal transduction

#### 3.5.1 Regulation of metabolites on pro-inflammatory and anti-inflammatory factors

*M. intestinale* interacts with the host immune system through its metabolites, thereby affecting the function of the gut-brain axis. The metabolites produced by *M. intestinale*, such as SCFAs and succinate, can modulate the expression levels of pro-inflammatory and anti-inflammatory factors (Figure 4A).

SCFAs, such as propionate and butyrate, have been shown to inhibit the production of pro-inflammatory factors (e.g., IL-6 and TNF-α) while promoting the expression of anti-inflammatory factors (e.g., IL-10) (Medina-Larqué et al., 2022; Xia et al., 2023). These metabolites exert their effects through various molecular mechanisms, such as GPR43/GPR41 receptor-mediated signaling pathways or epigenetic modifications that alter gene expression. Studies indicate that SCFAs can bind to G protein-coupled receptors (GPCRs) on intestinal epithelial cells, activating downstream signaling pathways to modulate immune responses (Medina-Larqué et al., 2022). Additionally, SCFAs can influence inflammatory responses by inhibiting HDAC activity, thereby altering gene expression patterns (Figure 3A).

These metabolites mitigate damage to the gut-brain axis by balancing immune responses and reducing excessive inflammation (Figure 4). For instance, Yang et al. found that total flavonoids from Chrysanthemum (TFC) can modulate the gut microbiota in AP model mice, increasing the abundance of *M. intestinale* and thereby influencing inflammatory responses (Yang et al., 2024).

#### 3.5.2 Transmits signals through the vagus nerve and HPA axis, affecting host stress and neuroinflammation

*M. intestinale* can transmit signals from the gut to the brain via the vagus nerve. The vagus nerve is a crucial component of the gut-brain axis, capable of bidirectional signal transmission, conveying gut information to the brain and relaying brain commands to the gut. In this process, key molecules or cell types, such as macrophages and dendritic cells, participate in signal transmission. Immune cells in the gut can detect metabolites of *M. intestinale* and transmit signals to the vagus nerve, ultimately influencing brain function (Figure 3A).

The HPA axis plays a crucial role in stress response. Studies have shown that the metabolites of *M. intestinale* can modulate the activity of the HPA axis, thereby alleviating chronic stress and neuroinflammation. Activation of the HPA axis leads to the release of stress hormones such as cortisol, and prolonged activation can result in chronic stress and neuroinflammation. By regulating the activity of the HPA axis, *M. intestinale* can mitigate the negative effects of chronic stress on the body (Figures 3, 4D–F).

Animal experimental data indicate that under conditions such as antibiotic intervention or fecal microbiota transplantation, differences exist in immune signal transduction. For example, antibiotic use alters the composition of the gut microbiota, thereby affecting the abundance and metabolic activity of *M. intestinale*, ultimately influencing immune signal transduction (Dobranowski et al., 2019; Hoque et al., 2022). Fecal microbiota transplantation can transfer healthy gut microbiota to recipients, restoring the balance of gut microbiota and improving immune function (Hoque et al., 2022; Figures 4D–F).

### 3.6 Protective effects of the intestinal barrier and blood-brain barrier

#### 3.6.1 Promote the expression of mucin and tight junction proteins

*M. intestinale* enhances the integrity of the intestinal barrier by increasing the expression of mucins (such as MUC2) and tight junction proteins (such as ZO-1 and occludin) in intestinal epithelial cells. Metabolites such as SCFAs play an important role in this process (Figure 4C).

Mucin is the main component of the intestinal mucus layer, forming a physical barrier that prevents direct contact between pathogens, harmful substances, and intestinal epithelial cells. Tight junction proteins are located between intestinal epithelial cells, forming a tight connection that prevents leakage of intestinal contents into the bloodstream. Experimental data show that increasing the expression of mucin and tight junction proteins can enhance the integrity of the intestinal barrier and reduce the leakage of pathogens and toxins (Figure 4C). Lee et al. found that citrus peel biotransformation products can improve intestinal inflammation and increase the mRNA level of occludin in Caco-2 cells (Lee et al., 2023).

#### 3.6.2 Reduce endotoxin leakage, thereby resisting systemic inflammation

*M. intestinale* can reduce the production and leakage risk of intestinal endotoxin (LPS), thereby decreasing systemic inflammatory responses. LPS is a component of the cell wall of Gram-negative bacteria, and when the intestinal barrier is compromised, LPS leaks into the bloodstream, triggering systemic inflammatory responses. Research shows that *M. intestinale* can enhance the integrity of the intestinal barrier, reducing LPS leakage and thus alleviating systemic inflammatory responses (Figure 4B).

The blood-brain barrier plays a crucial role in protecting the central nervous system from exogenous toxic substances. *M. intestinale* can further protect the brain from inflammatory damage by indirectly regulating the permeability of the blood-brain barrier. Preclinical research results indicate that *M. intestinale* can improve the function of the blood-brain barrier, reduce the entry of inflammatory factors into the brain, thereby alleviating neuroinflammation (Figure 3).

*M. intestinale* plays a multi-level role in the regulation of the gut-brain axis, including metabolism, immunity, and barrier protection. The synergistic effects of these mechanisms provide new perspectives for understanding the relationship between microbiota and host health, laying the foundation for subsequent discussions on disease associations and intervention strategies (Bang et al., 2023; Chen et al., 2025). For example, Chen et al.’s study demonstrated that HB alleviates PF by eliminating gut microbiota and metabolism, highlighting the feasibility of targeting IDO1 for PF treatment (Chen et al., 2025). Bang et al.’s research showed that the cardiolipin MiCL-1 isolated from *M. intestinale* has immunomodulatory effects and could serve as a potential therapeutic target (Bang et al., 2023).

However, the key limitation of this study is that our inference regarding *M. intestinale*’s influence on tryptophan metabolism is primarily based on community-level correlation data and known microbial metabolic knowledge, rather than direct functional validation of this specific species. Future research should isolate *M. intestinale* strains, assay their tryptophan metabolic capabilities *in vitro*, and conduct mono-colonization experiments in gnotobiotic animal models to conclusively determine its specific role in this pathway. Furthermore, While direct enzymological evidence for tryptophan metabolism by M. intestinale strains is currently lacking, its phylogenetic background and role as a key butyrate producer suggest it may influence intestinal tryptophan metabolic flux either directly or indirectly (by shaping the community ecology). Specifically, it may foster a microbial environment conducive to the generation of IPA over other indole derivatives, which aligns with its observed anti-inflammatory and neuroprotective potential. This hypothesis awaits future validation through tryptophan metabolite profiling of M. intestinale pure cultures.

## 4 Discussion

### 4.1 Potential association between *M. intestinale* and neuro-diseases

#### 4.1.1 Neuro-degenerative diseases

##### 4.1.1.1 Association between changes in *M. intestinale* abundance and β-amyloid deposition in Alzheimer’s disease models

Current research primarily focuses on observing changes in the abundance of *M. intestinale* in Alzheimer’s disease (AD) model animals and attempting to link it with β-amyloid (Aβ) deposition. Although specific experimental evidence remains relatively limited, some studies have provided preliminary correlative clues.

In Alzheimer’s disease mouse models, alterations in the gut microbiota are a common phenomenon, and *M. intestinale*, as a key member of the gut microbiota, has drawn significant attention regarding the relationship between its abundance changes and AD pathological features. Some studies have shown that in AD transgenic mouse models, the abundance of *M. intestinale* is significantly reduced, while Aβ deposition in the brain increases (Leigh et al., 2020). This suggests that the reduction of *M. intestinale* may be associated with impaired Aβ production or clearance.

However, the mechanism underlying this association remains unclear. One possible explanation is that metabolites of *M. intestinale* may indirectly influence Aβ deposition by affecting neuroinflammation. For instance, SCFAs, which are potential metabolites produced by *M. intestinale*, are known to have anti-inflammatory effects. If the abundance of *M. intestinale* decreases, reduced SCFA production may exacerbate neuroinflammation, thereby impairing microglial function and ultimately leading to decreased Aβ clearance efficiency. Conversely, whether increasing colonization by *M. intestinale* could reduce Aβ accumulation by boosting SCFA production is a question worthy of further investigation.

It is worth noting that reports on the relationship between *M. intestinale* abundance and AD pathology are not entirely consistent. Some studies have not found a significant association, which may be related to various factors, such as:

Host genetic background differences: Different strains of mice have different genetic backgrounds, which may affect the composition of the gut microbiota and the response to AD pathology.

Dietary intervention differences: Different dietary interventions may have varying effects on gut microbiota, thereby influencing study outcomes.

Disease stage: Changes in gut microbiota may vary at different stages of AD, which can also affect study outcomes.

Sample size: Some studies had small sample sizes, which may not detect subtle changes.

Therefore, when interpreting these findings, it is necessary to consider the differences in experimental design and conduct a cautious analysis. Future research should adopt more standardized experimental protocols and increase sample sizes to enhance the reliability of the results.

##### 4.1.1.2 Exploration as a potential protective factor or pathological marker

Given the important role of *M. intestinale* in the gut and its potential association with neurodegenerative diseases, researchers have begun to explore whether it could serve as a potential protective factor or pathological marker for AD.

In terms of protective effects, some evidence suggests that *M. intestinale* may delay neurodegenerative diseases through multiple pathways:

Anti-inflammatory effect: *M. intestinale* may inhibit neuroinflammation by producing metabolites with anti-inflammatory effects, such as SCFAs, thereby protecting neurons (Chen et al., 2025).

Maintaining intestinal barrier integrity: *M. intestinale* may help maintain the integrity of the intestinal barrier, reducing the entry of harmful substances from the gut into the bloodstream, thereby alleviating systemic inflammation and protecting the blood-brain barrier (Lee et al., 2023).

Regulating the immune system: *M. intestinale* may protect neurons by modulating immune system function and reducing attacks on the nervous system (Wu et al., 2026).

As a potential biomarker for early disease diagnosis, changes in the abundance of *M. intestinale* may reflect early pathological processes of the disease. If the risk of AD can be detected early by measuring the abundance of *M. intestinale* in feces, it would provide a valuable time window for prevention and treatment. However, current clinical data limitations include:

Insufficient cohort size: Existing clinical studies typically have small sample sizes, which are inadequate for fully evaluating the reliability of *M. intestinale* as a diagnostic marker.

Lack of longitudinal studies: The absence of long-term tracking of individuals makes it difficult to determine the causal relationship between changes in *M. intestinale* abundance and disease progression.

Differences in detection methods: Variations in the detection methods used across studies may lead to difficulties in comparing results.

Therefore, before using *M. intestinale* as a clinical diagnostic indicator, larger-scale, longer-term clinical studies with standardized detection methods are required.

Additionally, it is noteworthy that views on the association of *M. intestinale* with disease are not entirely consistent. Some studies suggest that *M. intestinale* is not directly linked to the development of AD. For example, one study found no significant change in the abundance of *M. intestinale* in fecal samples from AD patients (Xia et al., 2023). This controversy may stem from:

Differences in experimental design: Variations in experimental design across studies, such as sample size, disease stage, dietary intervention, etc., may lead to inconsistent results.

Detection method sensitivity: Different detection methods may have varying sensitivities, leading to discrepancies in the detection results of *M. intestinale* abundance.

Microbial interactions: The gut microbiota is a complex ecosystem, and the role of *M. intestinale* may be influenced by other microbial communities.

Therefore, when evaluating *M. intestinale* as a potential protective factor or pathological marker for AD, it is necessary to comprehensively consider various factors and conduct in-depth research.

### 4.2 Regulation of mental disorders and cognitive functions

#### 4.2.1 Mechanism of improving depressive-like behaviors by elevating BDNF and 5-HT levels in the depression model

**FIGURE 5:**
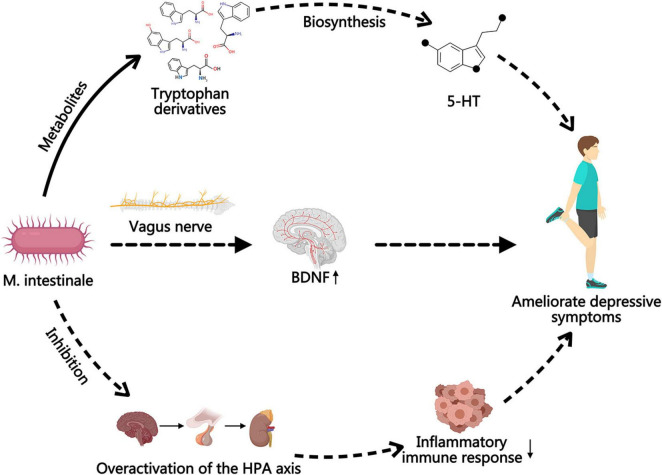
The regulatory mechanisms of *M. intestinale* in the gut-brain axis. **(A)** *M. intestinale* mediates succinate metabolism, modulates immune cell function, inhibits inflammatory cytokine release, and improves neurological function. **(B)** *M. intestinale* plays an important role in regulating IDO1-mediated ferroptosis, helping alleviate pulmonary fibrosis symptoms. **(C)** *M. intestinale potentially* regulates tryptophan metabolite levels→modulates neurotransmitters and derives butyrate mitigates skeletal muscle loss in cancer cachexia. **(D)** *M. intestinale* suppresses pro-inflammatory factor production and promotes anti-inflammatory factor expression via short-chain fatty acids. **(E)** *M. intestinale* modulates the vagus nerve’s influence on brain mental state. **(F)** *M. intestinale* mediates the functional mode of the HPA axis in gut-brain regulation.

In the field of depression research, the role of *M. intestinale* has gradually gained attention, particularly in how it influences depressive-like behaviors through neurotransmitters and neurotrophic factors. Studies have shown that *M. intestinale* can alleviate depressive symptoms through various mechanisms, with the elevation of brain-derived neurotrophic factor (BDNF) and serotonin (5-HT) levels being key components (Figure 5).

Critical pathway: The metabolites of *M. intestinale*, such as tryptophan derivatives, play a crucial role in regulating neurotransmitter synthesis. Tryptophan is a precursor for 5-HT synthesis, and 5-HT is an important neurotransmitter closely related to mood regulation. *M. intestinale* may promote 5-HT synthesis by influencing tryptophan metabolic pathways, thereby alleviating depressive symptoms (Zhang Z. et al., 2025; Figure 5).

Additionally, *M. intestinale* may also increase BDNF expression through vagus nerve activation. The vagus nerve is a crucial pathway connecting the gut and the brain, allowing gut microbiota to transmit signals to the brain and influence brain function. BDNF is an important neurotrophic factor essential for neuronal survival, growth, and differentiation. In individuals with depression, BDNF levels are typically lower, and increasing BDNF expression can alleviate depressive symptoms. Therefore, *M. intestinale*’s ability to enhance BDNF expression via vagus nerve activation may represent a significant antidepressant mechanism (Figure 5).

Behavioral evidence: In animal experiments, researchers commonly use behavioral tests such as the forced swim test and the tail suspension test to assess depressive-like behaviors in animals. In the forced swim test, animals are compelled to swim in water, and the duration of immobility is measured; longer immobility times indicate stronger feelings of despair and more pronounced depressive-like behaviors. In the tail suspension test, animals are suspended, and the duration of struggling is measured; shorter struggling times indicate stronger feelings of despair and more evident depressive-like behaviors.

Research has found that in mouse models of depression, reduced colonization of *M. intestinale* is associated with increased depressive-like behaviors. For example, one study found that in chronic stress model mice, the abundance of *M. intestinale* significantly decreased, while the immobility time of the mice in the forced swim test and tail suspension test increased, indicating aggravated depressive-like behaviors (Zhang et al., 2022). However, supplementing these mice with *M. intestinale* significantly reduced their immobility time, suggesting that *M. intestinale* supplementation can improve depressive-like behaviors.

Immune correlation: In addition to neurotransmitters and neurotrophic factors, *M. intestinale* may also influence the onset and progression of depression by modulating the immune system. Depression is closely associated with immune system dysfunction, such as the overactivation of the HPA axis, which is a key feature of depression. The HPA axis is a critical system for the body’s response to stress, and chronic overactivation can lead to elevated cortisol levels, thereby affecting brain function.

*M. intestinale* may alleviate depressive symptoms by suppressing the overactivation of the HPA axis and reducing inflammatory responses in the immune system. For example, some studies have found that supplementing with *M. intestinale* in depression model mice can lower serum cortisol levels, indicating its role in inhibiting excessive HPA axis activation (Shen et al., 2024; Figure 4D).

#### 4.2.2 Relationship between cognitive impairment, spatial memory deficits, and changes in microbiota

In addition to depression, *M. intestinale* is also associated with cognitive function, particularly spatial memory. Spatial memory refers to the ability to remember the location of objects in space, which is crucial for daily life.

Animal models: Researchers typically use the Morris water maze or novel object recognition test to assess animal cognitive function. In the Morris water maze test, animals must locate a hidden platform in a water pool, with the time and path taken to find the platform measured. Shorter search times and more direct paths indicate stronger spatial memory abilities in the animals. In the novel object recognition test, animals must distinguish between a new object and a familiar one, with the time spent exploring the new object measured. Longer exploration times of the new object suggest better cognitive abilities in the animals.

Research has found that the absence of *M. intestinale* may lead to impaired spatial memory. For example, one study found that germ-free mice showed a significant decline in spatial memory ability compared to mice colonized with *M. intestinale* (Leigh et al., 2020). This suggests that *M. intestinale* is crucial for maintaining normal spatial memory function.

Further research found that the effect of *M. intestinale* on spatial memory may be related to the synaptic plasticity of the hippocampus. Synaptic plasticity refers to the ability of changes in the strength of connections between neurons and is the basis of learning and memory. *M. intestinale* may improve cognitive function by regulating the synaptic plasticity of the hippocampus.

Microbiota-gut-brain axis: It remains unclear whether *M. intestinale* indirectly improves cognition by modulating gamma-aminobutyric acid (GABA)-ergic neurons or reducing neuroinflammation, but some studies have provided preliminary clues. GABA is an inhibitory neurotransmitter that can suppress neuronal excitability and is crucial for maintaining the balance of the nervous system. *M. intestinale* may enhance cognitive function by modulating the activity of GABAergic neurons.

Additionally, *M. intestinale* may indirectly improve cognition by reducing neuroinflammation. Neuroinflammation refers to the inflammatory response occurring in the nervous system, which can lead to neuronal damage and dysfunction, thereby affecting cognitive function. *M. intestinale* may produce anti-inflammatory metabolites that reduce neuroinflammation, thereby protecting neurons and improving cognitive function.

### 4.3 Metabolic diseases and energy balance

#### 4.3.1 The impact of reduced *M. intestinale* on insulin resistance and cognitive function under obesity and high-fat diet

The role of *M. intestinale* in metabolic diseases and energy balance, particularly its indirect effects on cognitive function under conditions of obesity and high-fat diet, is a current research hotspot.

Preclinical data: Studies have shown that in high-fat diet mouse models, decreased *M. intestinale* abundance often coincides with insulin resistance and impaired brain energy metabolism. A high-fat diet can lead to gut microbiota dysbiosis, significantly reducing the abundance of *M. intestinale*, a beneficial bacterium. Concurrently, high-fat diets induce insulin resistance, reducing the body’s sensitivity to insulin and disrupting blood sugar regulation. Additionally, high-fat diets cause brain energy metabolism disorders, impairing normal neuronal function.

Mechanism chain: A possible mechanistic chain regarding how *M. intestinale* affects insulin resistance and cognitive function is that its metabolites, such as succinate, may improve intestinal permeability, reduce systemic inflammation, and thereby protect blood-brain barrier function. A high-fat diet can increase intestinal permeability, allowing harmful gut substances to enter the bloodstream and trigger systemic inflammation. Systemic inflammation further damages the blood-brain barrier, leading to neuroinflammation and subsequently impairing normal neuronal function.

The metabolites of *M. intestinale* may help improve intestinal permeability and reduce systemic inflammation, thereby protecting the function of the blood-brain barrier. For example, succinic acid is an important metabolic intermediate with anti-inflammatory effects that can reduce intestinal inflammatory responses, thereby improving intestinal permeability (Figure 4C).

Cognitive outcomes: The regulatory effects of *M. intestinale* on synaptic proteins (such as PSD-95) expression or mitochondrial function may be closely related to cognitive function. Synaptic proteins are essential components of neuronal connections, crucial for maintaining normal neuronal function. Mitochondria serve as the energy factories of cells, providing energy for neurons. *M. intestinale* may improve cognitive function by modulating synaptic protein expression or mitochondrial function.

For example, PSD-95 is an important synaptic protein involved in synapse formation and stabilization. Studies have found that in AD model mice, the expression of PSD-95 is significantly decreased, while supplementation with *M. intestinale* can increase PSD-95 expression, thereby improving cognitive function (Maier et al., 2020).

Additionally, mitochondrial dysfunction is a significant feature of AD. Studies have found that mitochondrial function is significantly reduced in the brain tissues of AD patients, leading to insufficient energy supply to neurons. *M. intestinale* may improve cognitive function by enhancing mitochondrial function and providing energy to neurons.

### 4.4 Comparison of animal models with clinical data

#### 4.4.1 Similarities, differences, and challenges in translation between model construction, experimental data, and some clinical studies

Animal models have played a crucial role in studying the association between *M. intestinale* and diseases, but translating conclusions from animal experiments to clinical applications faces numerous challenges, requiring critical evaluation of their feasibility.

Species differences: The colonization rate, abundance, and function of *M. intestinale* in the intestines of mice and humans exhibit significant differences. In mice, *M. intestinale* is typically a key member of the gut microbiota, whereas in humans, its abundance may be lower or even absent in some individuals. Additionally, the functions of *M. intestinale* in the intestines of mice and humans may also differ.

Therefore, when extrapolating conclusions from animal experiments to humans, these species differences need to be taken into account. Human data may rely more on metagenome-wide association studies, analyzing genomic data from large populations to identify associations between *M. intestinale* and diseases.

Clinical localized: Current clinical studies have small sample sizes and employ single intervention methods, such as only testing fecal microbiota, lacking cerebrospinal fluid or imaging evidence. Fecal microbiota can only reflect the composition of gut microbiota, while cerebrospinal fluid and imaging evidence can more directly reflect the functional state of the brain. Therefore, when assessing the association of *M. intestinale* with diseases, it is necessary to integrate multiple data sources.

Conversion strategy: Future research needs to incorporate humanized mouse models or longitudinal cohorts to validate mechanisms. Humanized mouse models involve transplanting human gut microbiota into germ-free mice, thereby constructing an animal model that more closely resembles the human intestinal environment. Longitudinal cohorts refer to studies that track individuals over an extended period, allowing for a more accurate assessment of the causal relationship between changes in *M. intestinale* abundance and disease progression.

In summary, although animal models provide valuable clues for studying the association between *M. intestinale* and diseases, translating these findings into clinical applications still faces numerous challenges. Future research must overcome these challenges and adopt more scientific methods to truly reveal the role of *M. intestinale* in human health.

### 4.5 Research methods and technical challenges

#### 4.5.1 Animal model construction and validation methods

##### 4.5.1.1 Application of germ-free mice, antibiotic pretreatment, and fecal microbiota transplantation techniques

**FIGURE 6:**
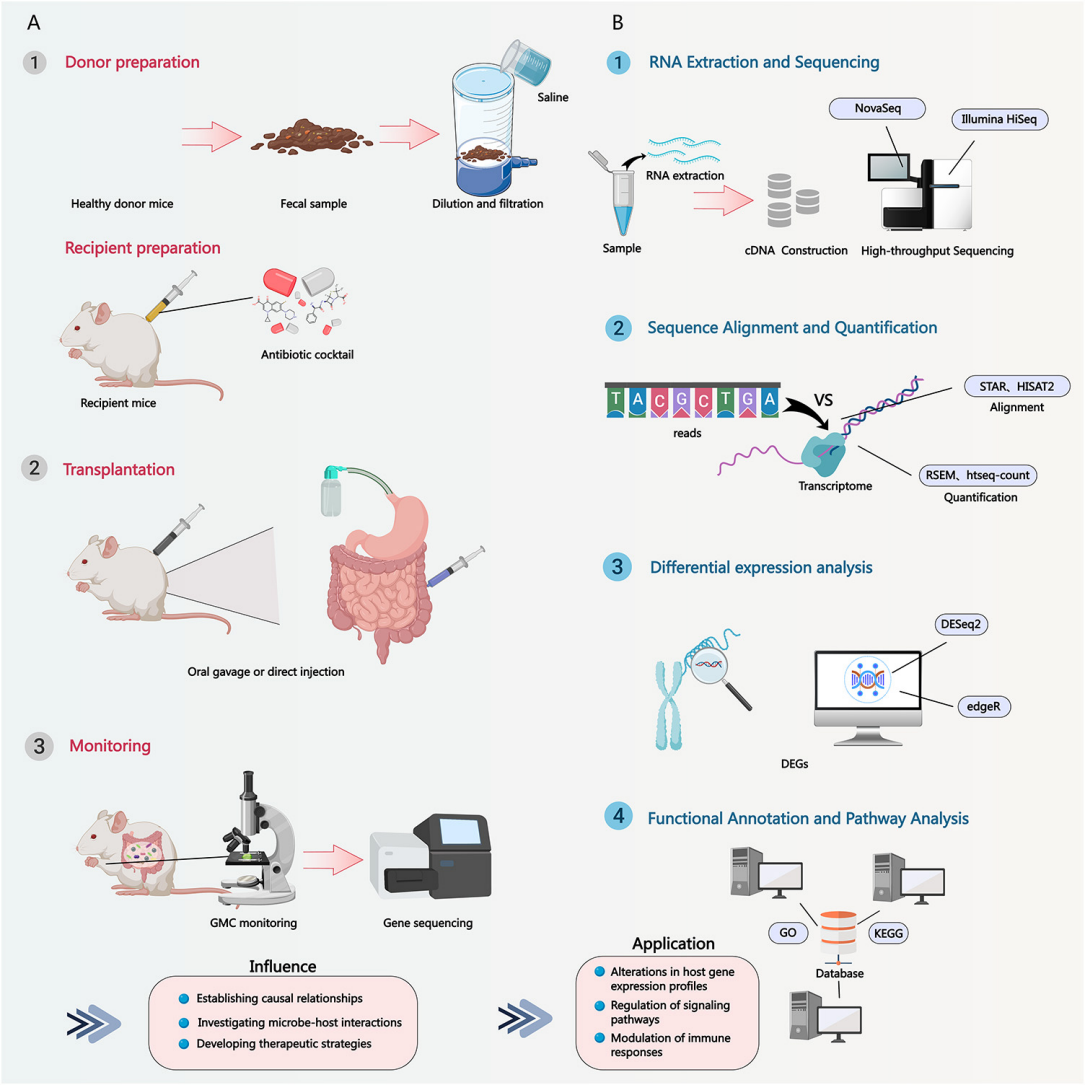
Transcriptomic analysis workflow and applications of *M. intestinale* and the feasible operation of related fecal microbiota transplantation techniques. **(A)** Feasible operational steps for *M. intestinale* fecal microbiota transplantation. **(B)** Overview of *M. intestinale* transcriptomic analysis strategies.

Germ-free mice, also known as axenic animals, refer to mice raised in a completely sterile environment with no detectable microorganisms inside or outside their bodies. The establishment of this model is crucial for studying *M. intestinale* because (Figure 6):

(1)    Studying the specific role of microorganisms: Germ-free mice provide a “blank” biological system, allowing researchers to introduce specific microorganisms (such as *M. intestinale*) and observe their individual effects on the host’s physiology, immune system, and nervous system. This eliminates interference from other microorganisms, enabling a more accurate assessment of *M. intestinale*’s function.
(2)    Revealing the regulatory mechanisms of the gut-brain axis: Research on the gut-brain axis requires distinguishing the complex interactions between microbes and the host. Germ-free mouse models allow researchers to study how *M. intestinale* directly or indirectly affects brain function and behavior through metabolites, immune regulation, or neural signaling without interference from other microbes.
(3)    Disease model construction: By colonizing *M. intestinale* in germ-free mice, specific disease states such as neurodegenerative diseases, mental disorders, or metabolic disturbances can be simulated. This facilitates the study of the role of *M. intestinale* in these diseases and the evaluation of potential therapeutic interventions.

Antibiotic pretreatment is another commonly used method to influence the composition of gut microbiota in specific research designs. The specific approach typically involves administering broad-spectrum antibiotics such as ampicillin, vancomycin, neomycin, and metronidazole to mice orally or via injection. The necessity and efficacy of antibiotic pretreatment are reflected in Figure 6A:

(1)    Elimination of the original microbiota: Antibiotics can significantly reduce or even eliminate the original microorganisms in the mouse gut, creating conditions for the colonization of specific microbial communities in subsequent studies. This helps researchers control the composition of gut microbiota and reduce experimental background noise.
(2)    Altering the microbiota structure: Antibiotic pretreatment changes the structure of the gut microbiota, reduces microbial diversity, and makes it easier for researchers to observe the colonization and growth of specific microorganisms (such as *M. intestinale*).
(3)    Enhancing colonization of specific bacterial populations: By selectively using antibiotics, the growth of certain bacteria can be suppressed, thereby creating a more favorable environment for the colonization of *M. intestinale*. For example, if *M. intestinale* is resistant to a certain antibiotic, pretreatment with that antibiotic can promote its colonization in the gut.

Fecal microbiota transplantation (FMT) technology is widely used in mouse studies, and its main implementation steps and precautions include (Figure 6A):

(1)    Donor selection and preparation: Select healthy donor mice, typically requiring microbiome analysis to ensure their gut microbiota composition meets experimental requirements. Collect fresh feces from donor mice and process them under sterile conditions, such as dilution with sterile saline and filtration.
(2)    Preparation of recipient mice: Recipient mice typically require antibiotic pretreatment to eliminate or reduce their own gut microbiota, creating a niche for the transplanted microbiota. The duration of pretreatment and the choice of antibiotics need to be optimized based on the experimental objectives.
(3)    Transplantation process: The treated fecal suspension is administered into the intestinal tract of recipient mice via gavage or direct injection. The dose and frequency of transplantation should also be adjusted according to the experimental design.
(4)    Post-transplantation monitoring: After transplantation, it is necessary to regularly monitor the gut microbiota composition of recipient mice to assess the success rate of transplantation and the stability of the microbiota. Analysis can be performed using methods such as 16S rRNA gene sequencing and metagenomic sequencing.

FMT technology significantly impacts research outcomes by reconstructing specific microbiomes (Figure 6A):

(1)    Verification of causal relationships: By transplanting specific microbial communities (including *M. intestinale*) from disease model mice to healthy mice, the causal role of these microbial communities in disease development can be verified. If the healthy mice develop similar disease phenotypes after transplantation, it indicates that these microbial communities play an important role in the disease.
(2)    Studying microbial interactions: FMT technology can be used to investigate interactions between different microbial communities. For example, *M. intestinale* can be co-transplanted with other specific microbiota into germ-free mice to observe their synergistic or antagonistic effects, as well as their impact on host health.
(3)    Development of therapeutic strategies: FMT technology itself can also serve as a treatment method. By transplanting fecal microbiota from healthy individuals into patients, it is possible to reconstruct the patient’s intestinal microecology, thereby treating diseases associated with microbiota dysbiosis.

#### 4.5.2 Multi-omics integration analysis strategy

##### 4.5.2.1 Integrated analysis of metagenomics, transcriptomics, and metabolomics

Metagenomic analysis is a technique that involves extracting DNA directly from environmental samples and then performing high-throughput sequencing. Its basic principle is (Figure 6B):

(1)    DNA extraction and sequencing: Extract DNA from all microorganisms in samples such as feces and intestinal contents, construct a DNA library, and perform high-throughput sequencing. Sequencing platforms include Illumina, PacBio, etc., which can generate large amounts of DNA sequence data.
(2)    Sequence Analysis and Species Identification: Align the obtained sequences with known microbial genome databases to identify the microbial species present in the sample and their relative abundance. Commonly used analytical tools include MetaPhlAn and Kraken.
(3)    Functional prediction: Through functional annotation of metagenomic data, the potential metabolic functions and gene functions of microbial communities are predicted. For example, the abundance of genes related to carbohydrate metabolism, amino acid metabolism, etc., in samples can be analyzed to infer the metabolic capabilities of microbial communities.

Metagenomic analysis is used to detect and describe the relative abundance and diversity of *M. intestinale* in samples, with methods including (Figure 6B):

(1)    Species abundance analysis: Through metagenomic sequencing, the DNA sequence quantity of *M. intestinale* in the sample can be directly determined, thereby ascertaining its relative abundance. For example, tools such as MetaPhlAn can be used to quantify the abundance of *M. intestinale* based on unique marker genes.
(2)    Diversity analysis: Metagenomic data can be used to assess the overall diversity of microbial communities in samples, including alpha diversity and beta diversity. Alpha diversity describes the species richness and evenness within a single sample, while beta diversity compares the differences in species composition between different samples.
(3)    Strain-level analysis: Metagenomic analysis can provide higher resolution than 16S rRNA gene sequencing, enabling the differentiation of various *M. intestinale* strains. This is of significant importance for studying the functional differences and ecological adaptability of different strains.

Transcriptome analysis is a technique used to understand gene expression regulation and cellular functional states by studying the transcriptional activity of all genes in specific cells or tissues. The process includes (Figure 6B):

(1)    RNA extraction and sequencing: Total RNA was extracted from the samples, and after quality assessment, cDNA library construction and high-throughput sequencing were performed. Commonly used sequencing platforms include Illumina HiSeq and NovaSeq.
(2)    Sequence alignment and quantification: Align the obtained RNA sequences (reads) to the reference genome or transcriptome to determine the expression level of each gene. Commonly used alignment tools include STAR and HISAT2, while quantification tools include RSEM and htseq-count.
(3)    Differential expression analysis: Compare gene expression differences between different treatment groups to screen out differentially expressed genes (DEGs). Commonly used analysis tools include DESeq2, edgeR, etc.
(4)    Functional annotation and pathway analysis: Perform functional annotation on differentially expressed genes to understand the biological processes and signaling pathways they are involved in. Commonly used databases include GO (Gene Ontology) and KEGG (Kyoto Encyclopedia of Genes and Genomes).

Application of transcriptome analysis in elucidating the impact of *M. intestinale* on host gene expression:

(1)    Changes in host gene expression profiles: By comparing the transcriptome data of intestinal, brain, and other tissues between mice colonized with *M. intestinale* and control mice, the impact of *M. intestinale* on host gene expression can be understood. For example, it can be observed whether *M. intestinale* affects the expression of genes related to inflammation or neurotransmitter synthesis.
(2)    Signal pathway regulation: Transcriptome analysis can reveal how *M. intestinale* affects host physiological functions by regulating specific signaling pathways. For example, studies can investigate whether *M. intestinale* activates or inhibits inflammatory pathways such as NF-κB and MAPK, or influences neurotransmitter-related pathways like BDNF and 5-HT (Figures 3, 5).
(3)    Immune response regulation: By analyzing the transcriptome data of immune cells, it is possible to understand how *M. intestinale* affects the host’s immune response. For example, it can be observed whether *M. intestinale* regulates the activation, differentiation, and function of immune cells such as T cells and B cells.

Metabolomics is a technique for comprehensive analysis of all small molecule metabolites in biological samples. Its integrated approaches include:

(1)    Metabolite extraction: Extract metabolites from biological samples (such as feces, serum, tissues, etc.). Common extraction methods include liquid-liquid extraction, solid-phase extraction, etc., and appropriate extraction methods should be selected according to different types of metabolites.
(2)    Instrumental analysis: High-resolution mass spectrometry (such as LC-MS, GC-MS) is used to separate and detect extracted metabolites. Different mass spectrometry techniques are suitable for different types of metabolites.
(3)    Data processing and quantification: Preprocess mass spectrometry data (e.g., denoising, correction, normalization), followed by metabolite identification and quantification. Commonly used databases include HMDB (Human Metabolome Database) and KEGG.
(4)    Statistical analysis and pathway analysis: Use statistical methods (such as PCA, PLS-DA) to analyze metabolic differences between different treatment groups, and we will further perform pathway enrichment analysis to understand the biological significance of metabolite changes.

The importance of metabolomics in understanding the function of *M. intestinale*:

(1)    Metabolite identification: Metabolomics can identify various metabolites produced by *M*. *intestinale*, such as SCFAs, amino acid derivatives, vitamins, etc. These metabolites may directly affect the physiological functions of the host (Li J. et al., 2024). For example, *M. intestinale* can produce succinate, propionate, and 3-hydroxybutyrate, among others (Murakami et al., 2024).
(2)    Metabolic pathway analysis: By analyzing metabolomic data, the metabolic pathways of *M. intestinale* can be understood, such as carbohydrate metabolism, amino acid metabolism, and lipid metabolism. This helps to understand how *M. intestinale* utilizes substrates and interacts with other microorganisms.
(3)    Host-microbe interaction: Metabolomics can reveal the metabolic interactions between *M. intestinale* and the host. For example, it can study how the metabolites of *M. intestinale* affect the host’s metabolic pathways, or how the host’s metabolites influence the growth and metabolism of *M. intestinale* (Figure 4).

The holistic perspective brought by the integration of metagenomics, transcriptomics, and metabolomics (Figure 6):

(1)    Causal relationship validation: Metagenomic analysis reveals the composition and functional potential of microbial communities, transcriptomic analysis reveals gene expression levels, and metabolomic analysis reveals changes in metabolites. The combination of the three can verify whether microbial gene expression is related to the production of metabolites, thereby validating causal relationships.
(2)    Functional mechanism analysis: Metagenomics can identify the presence of *M. intestinale* and its potential metabolic functions, transcriptomics can reveal the gene expression of *M. intestinale* under specific conditions, and metabolomics can identify the metabolites produced by *M. intestinale*. The combination of the three can provide an in-depth understanding of how *M. intestinale* affects the physiological functions of the host through specific metabolic pathways.
(3)    Biomarker discovery: Through multi-omics integration analysis, biomarkers associated with *M. intestinale* can be identified for disease diagnosis and prognosis evaluation. For example, by combining metagenomic, transcriptomic, and metabolomic data, *M. intestinale*-specific metabolites related to neurodegenerative diseases can be screened out.

Using this information to gain a deeper understanding of the biological functions of *M. intestinale* and its role in the gut-brain axis can (Figure 4):

(1)    Neurotransmitter modulation: *M. intestinale* may affect the synthesis and release of neurotransmitters such as serotonin by metabolizing substances like tryptophan, thereby influencing brain function and behavior (Figures 3, 4A).
(2)    Immunomodulation: *M. intestinale* may regulate the production of pro-inflammatory and anti-inflammatory factors by generating metabolites such as short-chain fatty acids (SCFAs), thereby influencing immune responses and neuroinflammation (Figures 4D,F).
(3)    Intestinal barrier protection: *M. intestinale* may enhance intestinal barrier function by promoting the expression of mucins and tight junction proteins, reducing endotoxin (LPS) leakage, thereby decreasing systemic inflammation (Figure 4C).

## 5 Conclusion

### 5.1 Limitations in mechanistic research

#### 5.1.1 Indirect causality and confounding factors

The predominant reliance on correlational analyses between microbial abundance and host phenotypes, without validation through mono-colonization in germ-free animal models, renders it impossible to distinguish whether *M. intestinale* is a direct driver of neurological effects or merely a bystander. Furthermore, its survival and function are embedded within complex cross-feeding networks, such as dependence on metabolites like succinate or hydrogen produced by other commensals. Consequently, isolating its independent effects from those mediated by the broader community remains a significant challenge. Additionally, host genetic background and dietary composition, particularly the ratio of dietary fiber to fat, are major determinants of *M. intestinale*’s abundance and metabolic output. This high degree of host heterogeneity and environmental influence severely limits the generalizability of proposed mechanisms across diverse populations.

#### 5.1.2 Evidence gaps in proposed metabolic pathways

Several key mechanisms attributed to *M. intestinale* currently lack direct evidence. While its abundance correlates with butyrate levels, direct measurement of butyrate concentration and its impact on blood-brain barrier permeability in mono-colonized hosts is absent. The hypothesis regarding its role in tryptophan metabolism and the production of IPA is primarily based on phylogenetic proximity to known IPA-producing Clostridia. However, critical evidence, such as the identification of functional tryptophanase gene clusters in its genome, quantitative measurement of IPA production, and demonstration of AhR activation is missing. Other potentially relevant pathways, like secondary bile acid metabolism through enzymes such as 7α-dehydroxylase, remain completely unexplored, with no genomic annotation or experimental validation in gnotobiotic models.

#### 5.1.3 Unclear neurosignaling transmission pathways

The precise routes through which *M. intestinale*-derived signals reach the central nervous system are poorly defined. It is unclear whether its metabolites, like butyrate, can directly activate enterochromaffin cells to release serotonin (5-HT) and subsequently stimulate vagal afferent nerves. Similarly, its potential immunomodulatory effects, such as regulating macrophages to release neuroactive cytokines like IL-22 and their subsequent impact on glial cells require rigorous investigation. Furthermore, direct experimental evidence is needed to determine whether *M. intestinale* metabolites can modulate the expression of tight junction proteins (e.g., Claudin-5, ZO-1) to influence blood-brain barrier permeability.

### 5.2 Deficiencies and challenges in translational development

#### 5.2.1 Functional heterogeneity at the strain level

Significant genomic variation among different *M. intestinale* isolates suggests potential functional divergence in key metabolic outputs. This strain-level heterogeneity poses a major risk for developing universal probiotic formulations, necessitating the precise identification and selection of “high-functioning” clinical strains. The lack of established genetic tools, such as targeted gene knockout or overexpression systems, further impedes functional validation through reverse genetics, forcing continued reliance on correlational data. From a bioprocessing perspective, its strict anaerobic requirements, slow growth kinetics (doubling time > 8 h), and sensitivity to oxygen present substantial challenges for cost-effective, large-scale fermentation and long-term storage stability.

#### 5.2.2 Limitations of preclinical models

Current animal models have inherent shortcomings for translating findings to humans. Physiological and immunological differences between murine and human guts, coupled with the naturally higher abundance of *M. intestinale* in humans, may limit the predictive value of rodent studies. While intestinal organoids offer a human-derived alternative, they lack the integrated neuro-immune-microbial microenvironment essential for modeling systemic gut-brain communication. Furthermore, most existing human cohort studies are biased toward Western populations with specific dietary habits, potentially overlooking functional nuances relevant to Asian or other demographic groups with distinct dietary patterns.

#### 5.2.3 Technical bottlenecks in formulation and delivery

Developing a viable biotherapeutic product faces multiple delivery hurdles. *M. intestinale* exhibits poor tolerance to low gastric pH and bile salts, which can drastically reduce viable cell delivery to the colon. Protective strategies, such as microencapsulation with acid-resistant polymers (e.g., alginate/chitosan) or co-administration with protective prebiotics (e.g., resistant starch), require optimization. Furthermore, established gut microbiota exert strong colonization resistance, necessitating strategies-like co-administration with specific prebiotics or genetic engineering for enhanced gut adherence to ensure durable engraftment. The absence of targeted, site-specific (e.g., colon) release systems further complicates achieving consistent and localized therapeutic effects.

#### 5.2.4 Safety concerns and regulatory ambiguity

A comprehensive safety profile for *M. intestinale* is currently lacking. Although considered commensal, its potential risk for opportunistic translocation and infection in immunocompromised hosts has not been systematically assessed. The long-term consequences of supplementation on host physiology, including potential disruptions to core metabolic processes like the bile acid pool or vitamin K metabolism, remain unknown due to a lack of chronic toxicology studies. From a regulatory standpoint, its classification is unclear-whether as a probiotic, a live biotherapeutic product (LBP), or a dietary supplement-creating significant uncertainty for the clinical development and approval pathway.

### 5.3 Integrated framework of limitations: from mechanism to application

The current landscape of *M. intestinale* research in the gut-brain axis is constrained by interconnected limitations across basic science and translational development. Mechanistically, weak causal evidence, unvalidated metabolic pathways, and poorly defined signaling routes hinder the construction of a reliable mechanistic network and the identification of clear therapeutic targets. Translationally, significant strain heterogeneity, poor predictive validity of preclinical models, suboptimal formulation technology, and unknown long-term safety profiles collectively result in protracted, high-risk product development cycles. The core challenge stemming from these combined gaps is the formidable difficulty in designing precise, evidence-based intervention strategies.

### 5.4 Prioritized future research directions

To address these limitations, a focused, multi-pronged research agenda is essential.

1. At the mechanistic level, priority should be given to establishing direct causality through mono-colonization experiments in germ-free animals, integrated with metabolomic and neurobehavioral analyses. Concurrently, coupling metagenome-assembled genomes (MAGs) with culturomics will facilitate the screening and isolation of “elite” strains with high butyrate or IPA production.
2. For translational advancement, developing genetic toolkits (e.g., CRISPR-based systems) to create reporter strains (e.g., for butyrate sensing) would enable real-time monitoring of bacterial activity *in vivo*. In parallel, engineering advanced delivery systems, such as mucosa-adhesive hydrogel microparticles, is crucial to overcome colonization barriers.
3. Clinically, initiating small-scale Phase I safety trials to assess tolerability and impact on host metabolite profiles is a critical first step. Ultimately, identifying patient stratification biomarkers, such as baseline intestinal fiber content or inflammatory status, will be key to enabling personalized, precision interventions.

By systematically acknowledging and addressing these limitations, future research can shift focus toward validating causal mechanisms, overcoming technical bottlenecks, and building a robust translational roadmap, thereby advancing *M. intestinale* from a promising subject of basic research toward tangible clinical application.
